# Supplementary material for: Aggressive natural killer-cell leukemia mutational landscape and drug profiling highlight JAK-STAT signaling as therapeutic target
Source: Nat Commun. 2018 Apr 19;9:1567. doi: 10.1038/s41467-018-03987-2 (PMC5908809; doi:10.1038/s41467-018-03987-2)
Supplement: Supplementary file 1 — Supplementary Information [file 41467_2018_3987_MOESM1_ESM.pdf]

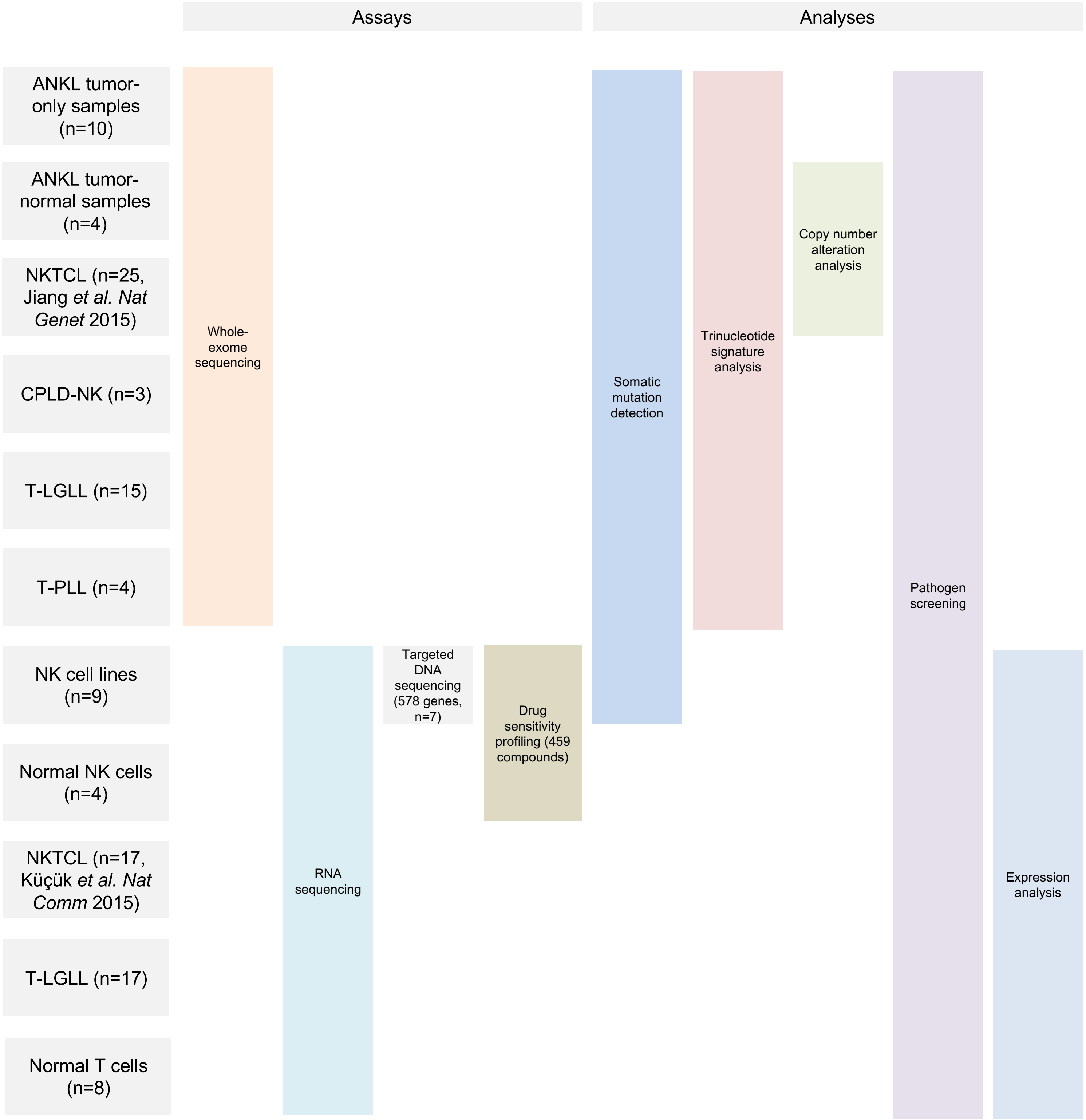

Supplementary Figure 1. Summary of samples, assay types and analyses included in the study. Rows of the table indicate sample cohorts listed on the left and columns indicate assay types or analyses performed on the samples.

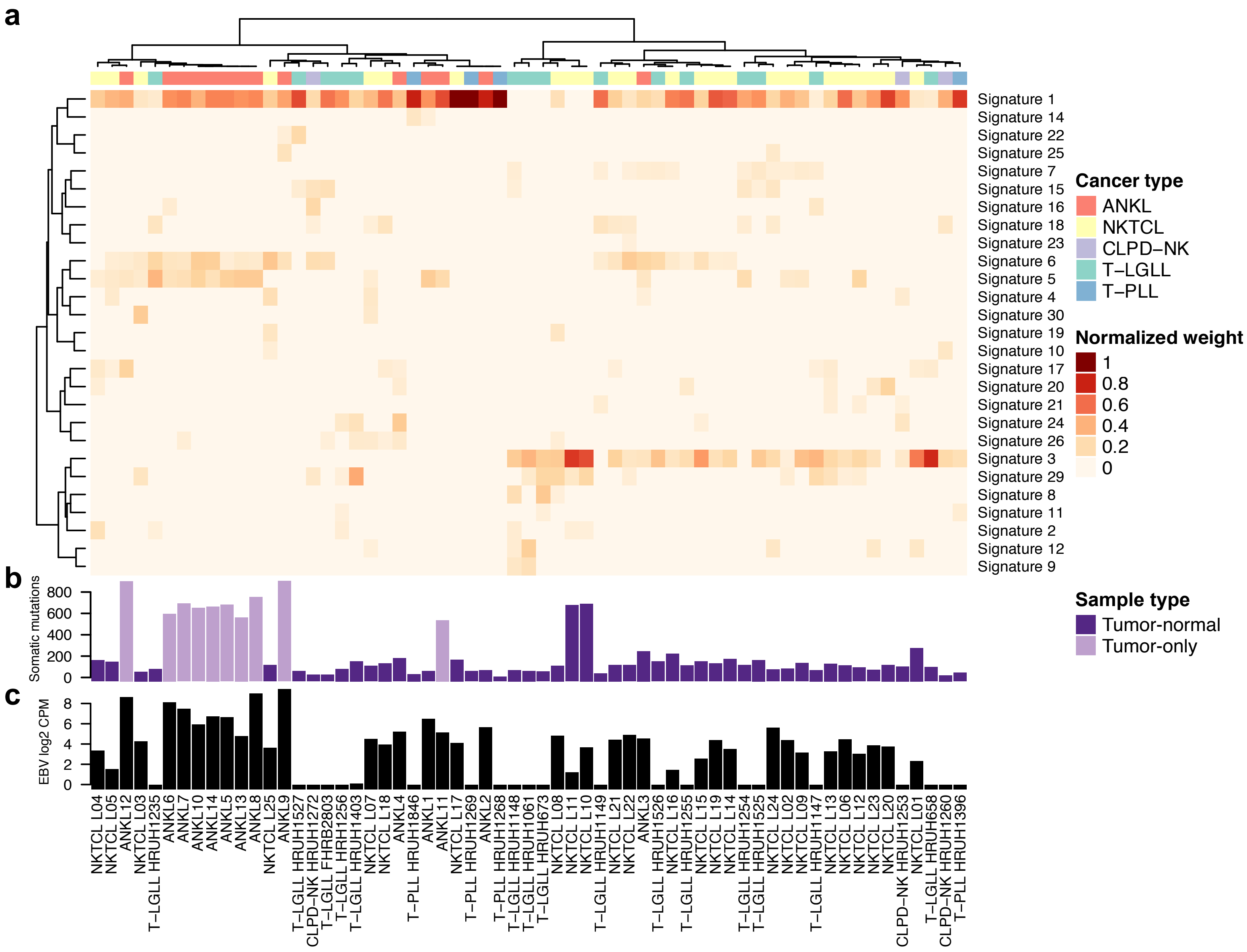

Supplementary Figure 2. Comparison of mutational signatures, mutation load and EBV genome reads in ANKL and other related cancers. (a) Heatmap showing normalized weights of mutational signatures identified using deconstructSigs. Signatures with zero weight in all samples are omitted. (b) Total somatic mutations, including synonymous mutations. (c) Reads mapping to the EBV genome as log2 counts per million (CPM) with 1 CPM added to all samples. Mutational signatures and somatic mutation load are based on variant calls including synonymous mutations. For tumor-only ANKL samples, the last stringent functional and population frequency-based filtering step (step 10, Supplementary Data 1) is omitted to increased comparability of the mutational signatures.

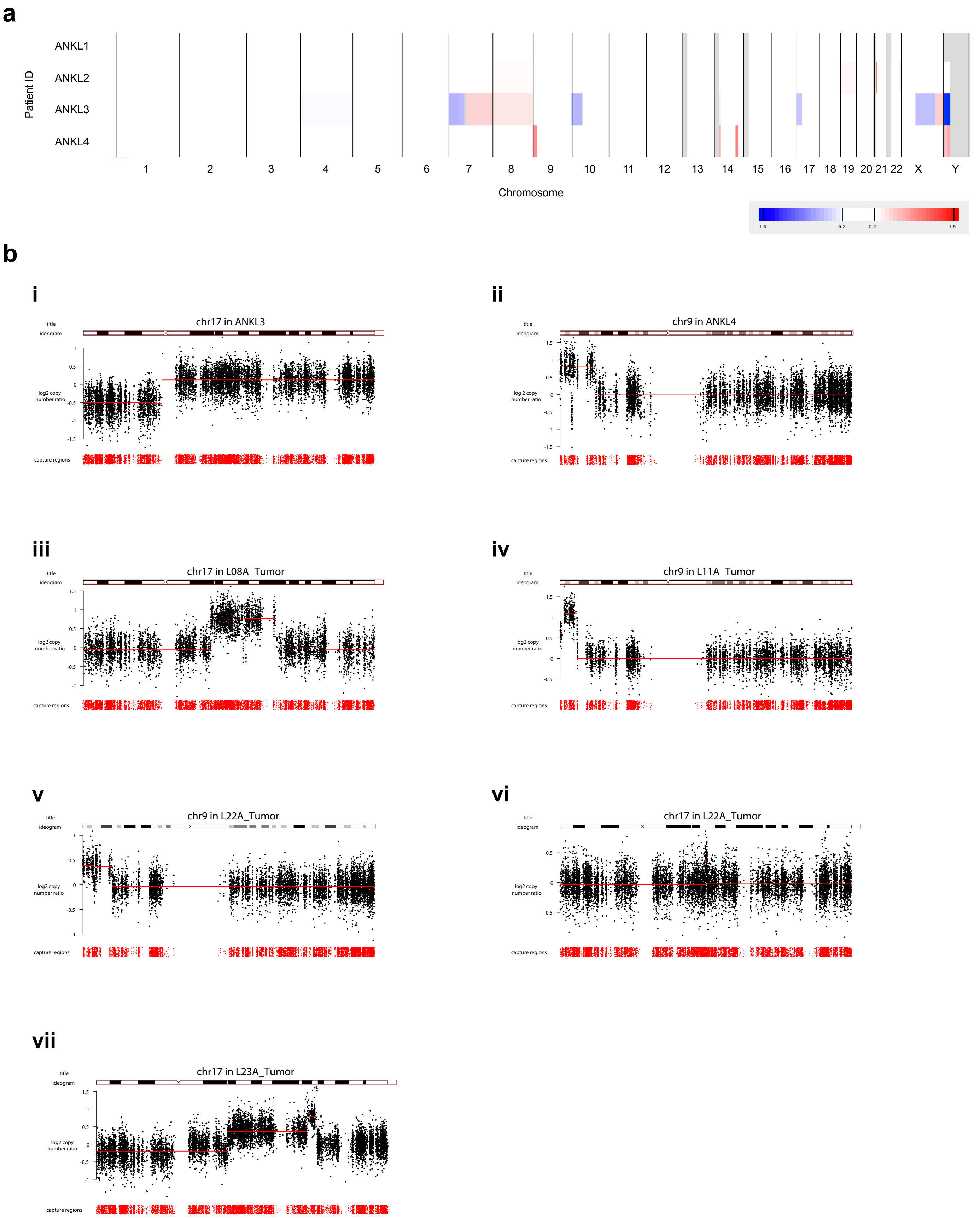

Supplementary Figure 3. Copy number alterations in ANKL and NKTCL patients. (a) Somatic copy-number alterations in 4 tumor-normal-paired ANKL samples. Copy-number alterations with $\log_2$ copy number ratios over 0.25 are shown. Color key indicates the $\log_2$ copy number ratio. (b) Graphs showing results of copy number alteration analysis based on WES data for selected cases. (i) 17p deletion containing *TP53* in patient ANKL3. ii) 9p24 gain containing *JAK2* in patient ANKL4. (iii) Gain of a region in 17q containing STAT3, STAT5A and STAT5B in NKTCL patient L08. (iv) 9p24 gain containing *JAK2* in NKTCL patient L11. (v) 9p24 gain containing *JAK2* in NKTCL patient L22. (vi) Gain of a region in 17q containing STAT3 in NKTCL patient L22. (vii) Gain of a region in 17q containing STAT3, STAT5A and STAT5B in NKTCL patient L23.

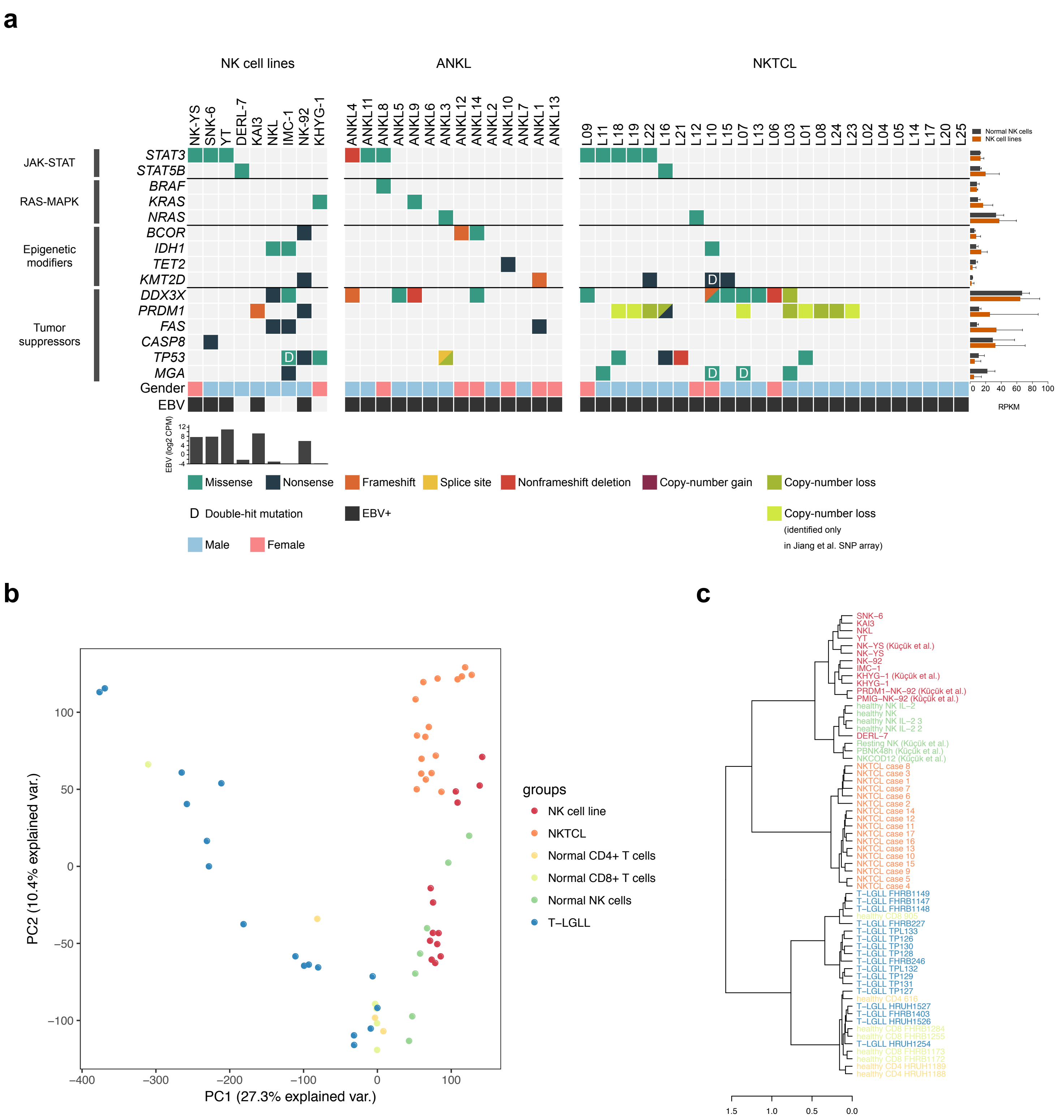

Supplementary Figure 4. Mutational and transcriptomic comparison of NK cell lines, primary lymphocytes, and primary NK-cell malignancies. (a) Selected functionally relevant mutations identified by RNA sequencing or targeted DNA sequencing in NK cell lines and by WES in ANKL and NKTCL. Diagonally split dual-colored rectangles indicate the presence of two mutations of different type in the same sample. RNA-seq reads mapping to the EBV genome are reported as counts per million (CPM) under the figure for the cell lines. Expression estimates of mutated genes in normal NK cells and NK cell lines are shown on the right as reads per kilobase per million mapped reads (RPKM), with bar length indicating mean and error bars representing range. Complete lists of mutations identified in the cell lines is available in Supplementary Data 6. (b) Principal component analysis (PCA) of expression estimate data from NK cell lines from this study (n=9) and from Küçük et al. (n=4), normal NK cells from this study (n=4) and from Küçük et al. (n=3), primary NKTCL samples from Küçük et al. (n=17), in-house T-LGL samples (n=17) and in-house normal CD4+ (n=3) and CD8+ (n=5) T cells. (c) Hierarchical clustering of expression estimate data using Spearman correlation distance and Ward's linkage. CPM data was used in PCA and hierarchical clustering analyses.

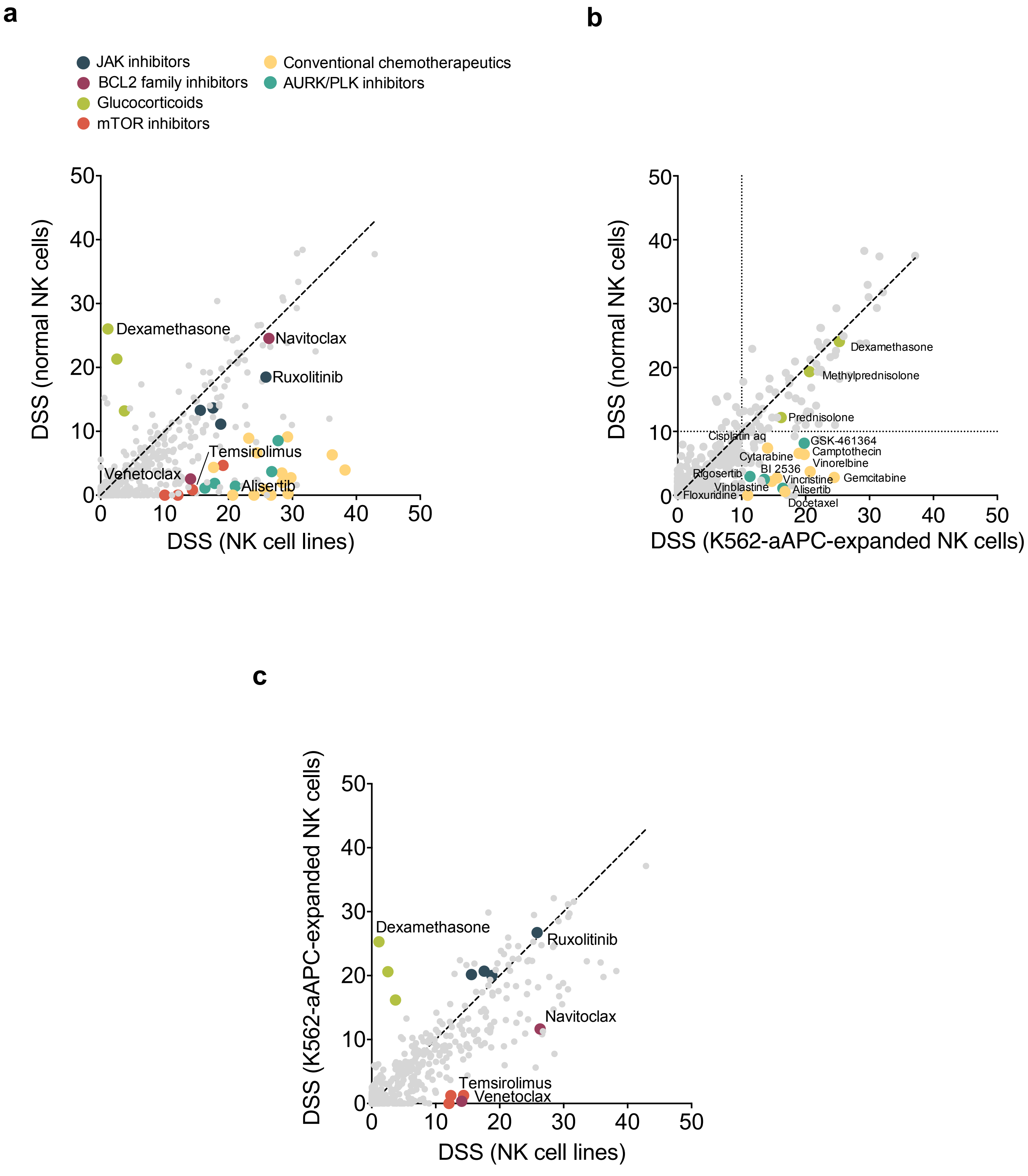

Supplementary Figure 5. Comparison of drug sensitivity profile of NK cell lines compared to normal NK cells. (a) Scatter plot comparing median DSS of NK cell lines (n=9) to normal primary NK cells (n=3). (b) Scatter plot comparing median DSS of K562-aAPC-expanded proliferating normal NK cells (n=1) to normal (not actively proliferating) primary NK cells (n=3). Both cell types were cultured in the presence of IL-2. (c) Scatter plot comparing median DSS of NK cell lines (n=9) to K562-aAPC-expanded proliferating normal NK cells (n=1).

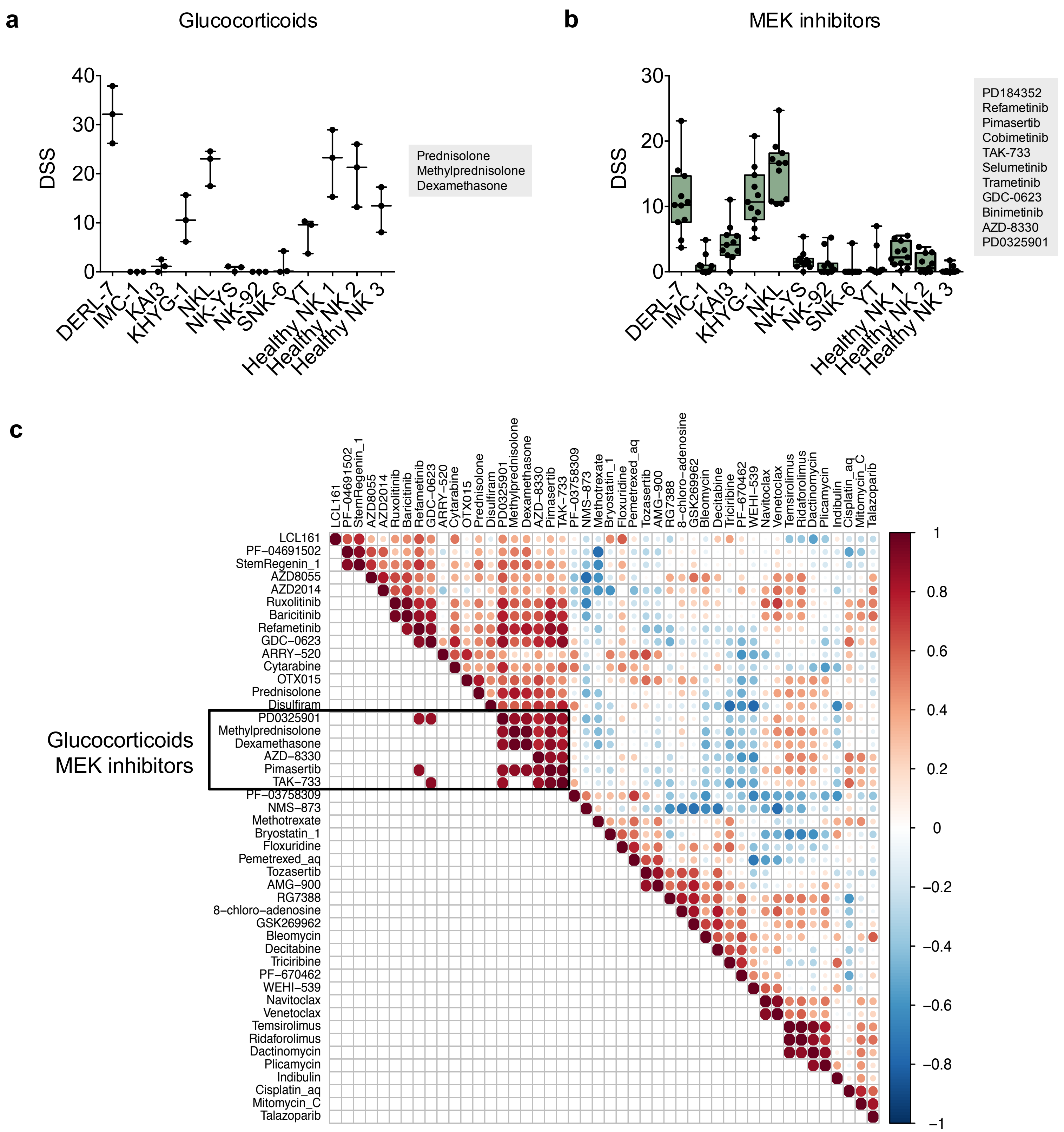

Supplementary Figure 6. Drug responses to MEK inhibitors and glucocorticoids in NK cells. (a) DSS of glucocorticoids in NK cell lines and normal NK cells. Each dot indicates the DSS of a glucocorticoid in the figure. (b) DSS of MEK inhibitors in NK cell lines and normal NK cells. Each dot indicates the DSS of a MEK inhibitor listed in the figure. Horizontal lines indicate median, error bars indicate range, and boxes represent interquartile ranges. (c) Visualization of correlations between DSS of the 10% of compounds with most variable DSS across the cell lines (n=9). Point color and size indicate Spearman's correlation coefficient, with color according to the color scale on the right and larger point indicating higher coefficient. Upper right half on the graph includes all correlation coefficients and lower left half ones significant at a FDR of 15%. Drugs are organized by hierarchical clustering using the complete linkage method and Euclidean distance. The cluster containing glucocorticoids and MEK inhibitors is highlighted by a rectangle.

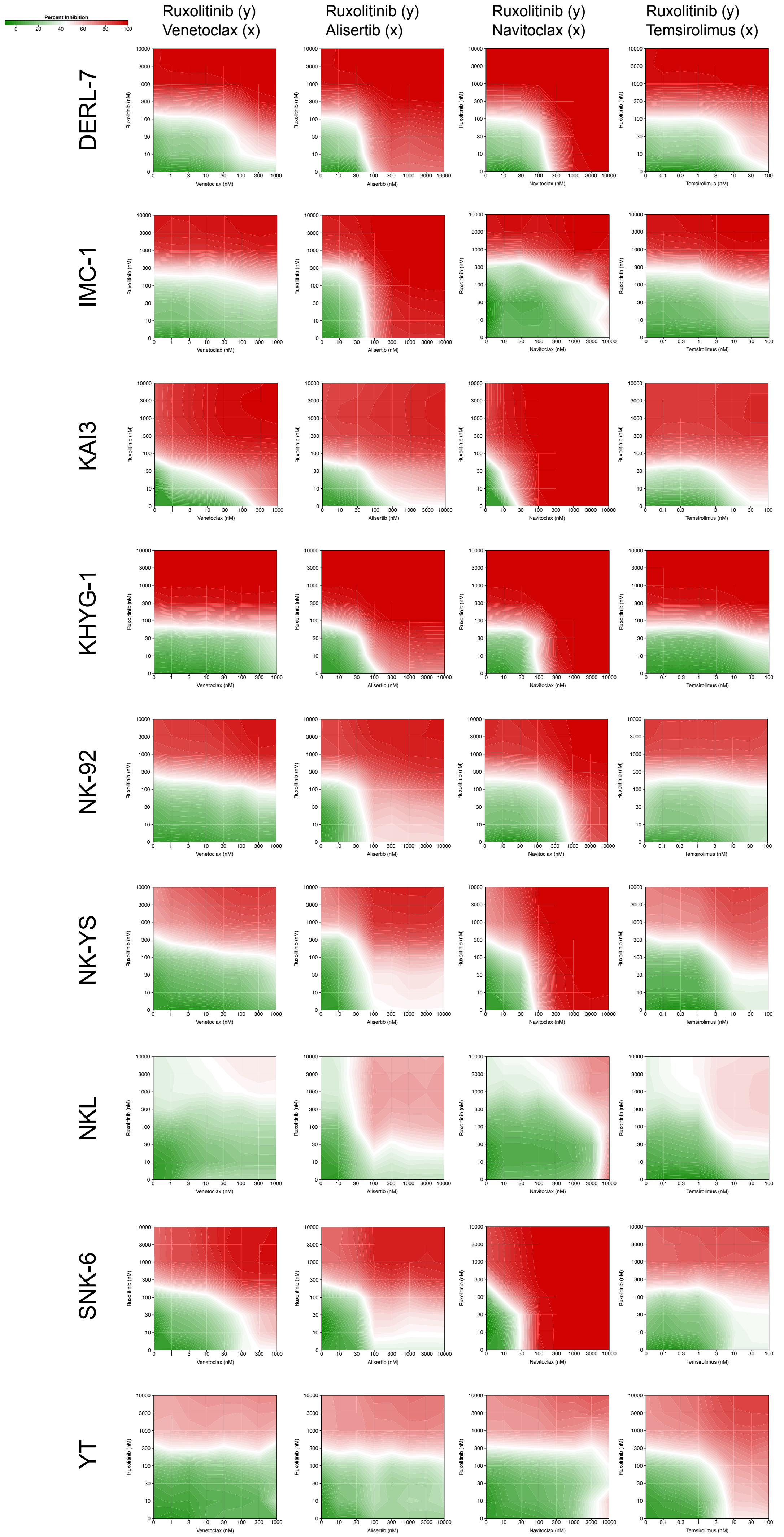

Supplementary Figure 7. Drug combination sensitivity landscapes in NK cell lines. Heatmaps showing inhibition percentages achieved at indicated doses of drug combinations. Shown are representative results from one out of two experiments.

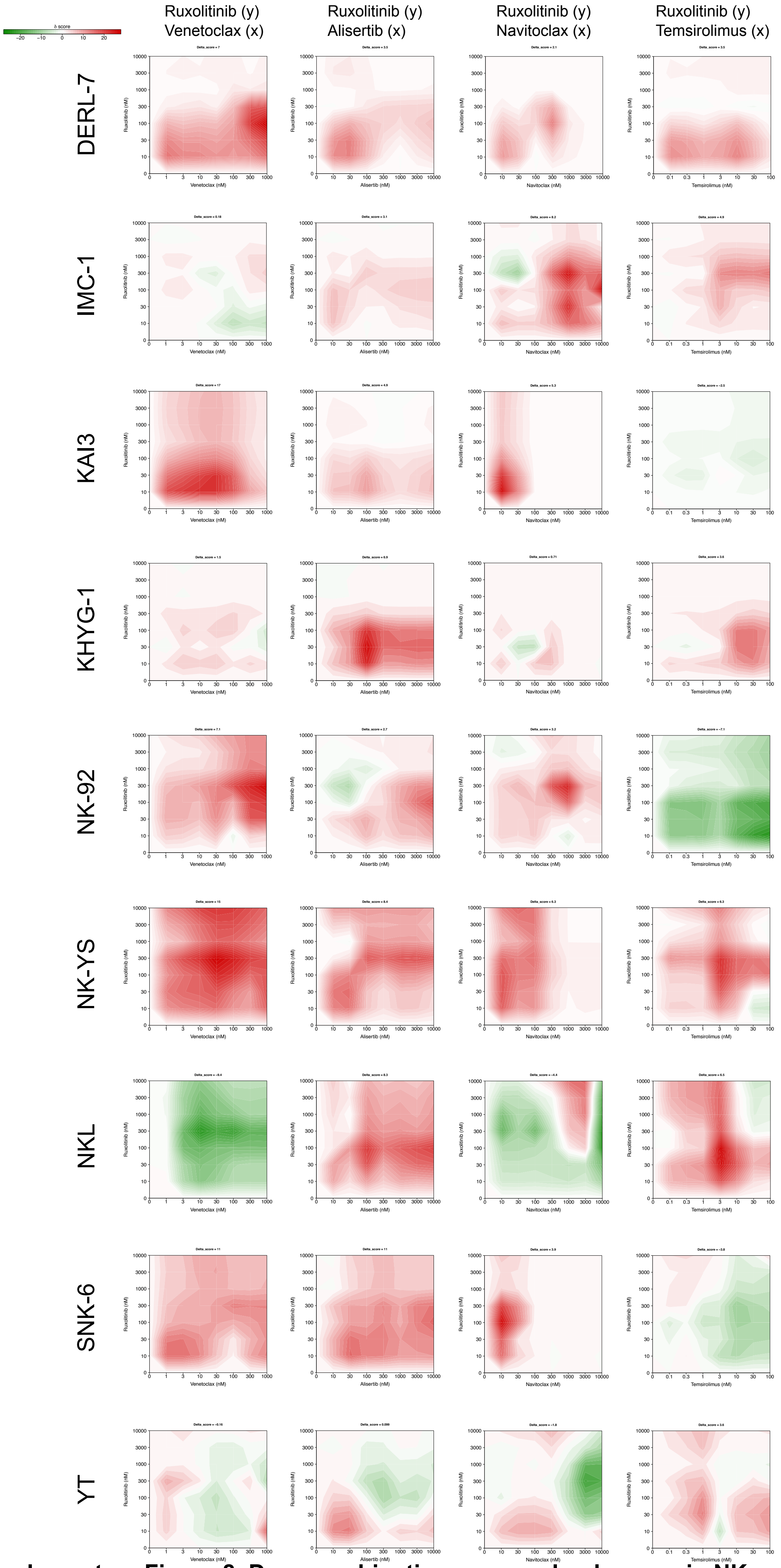

Supplementary Figure 8. Drug combination synergy landscapes in NK cell lines. Heatmaps showing delta synergy scores achieved at indicated doses of drug combinations. Shown are representative results from one out of two experiments.

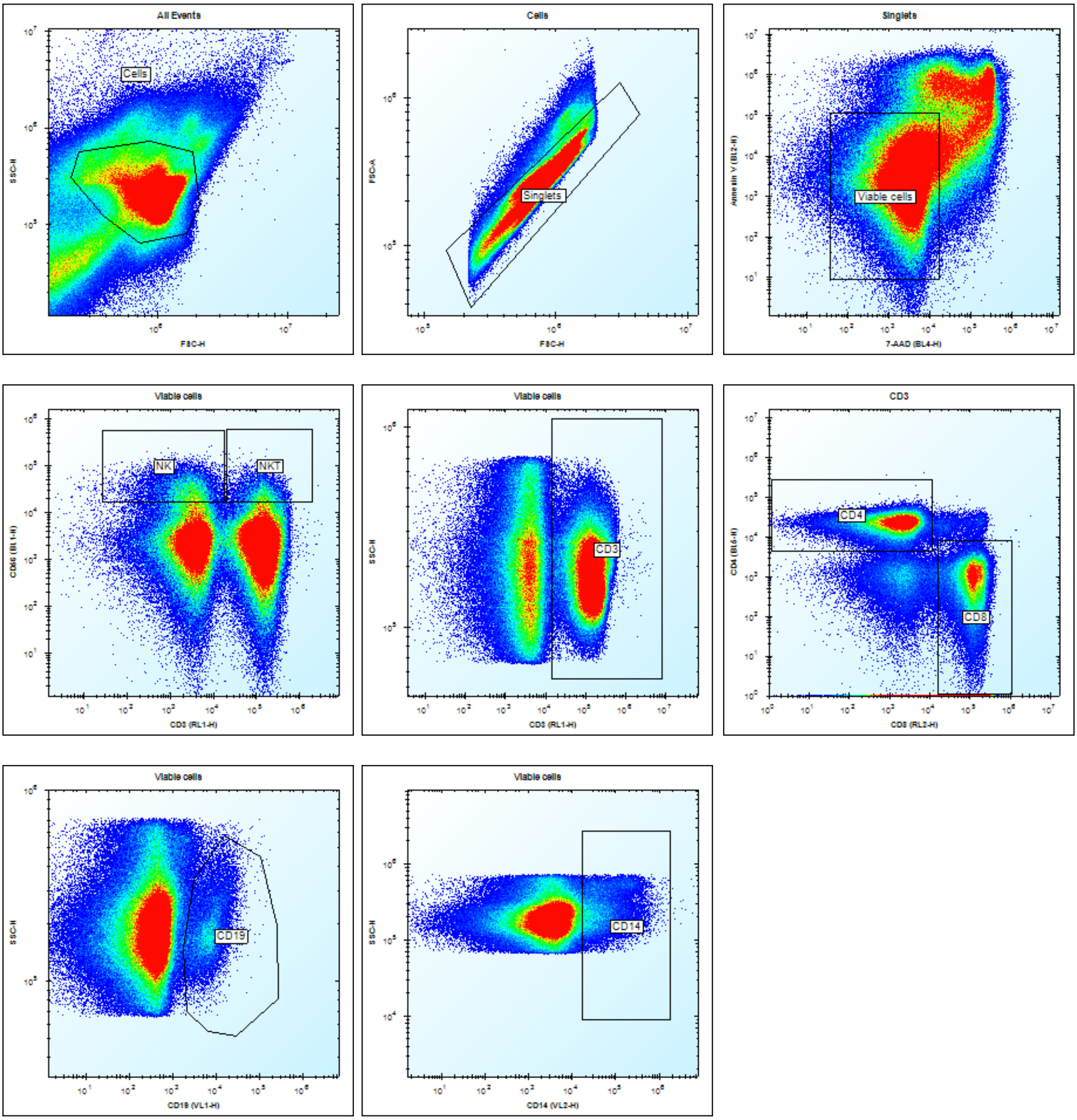

Supplementary Figure 9. Gating strategy in flow cytometry-based drug sensitivity assay.

Supplementary Table 1. Clinicopathological characteristics of 14 ANKL patients.

						Sites of involvement									Immunophenotype at diagnosis (% positive cells or pos/neg)**									
Patient ID	Gender	Age	Ethnicity	Year of diagnosis	Status	BM	PB	Spleen	Liver	Skin	Nasal	Previous history of NKTCL	EBV	EBV detection method	CD2	CD3	CD7	CD16	CD56	CD57	TCR rearrangement	Known cytogenetic abnormalities	Nakashima/Karube et al. ID	DNA source
ANKL1	F	22	Japanese	2004	DoD	yes	yes	yes	yes	no	no	yes*	positive	PCR	98	29	90	2	76	9	TCRb negative (southern blotting)	46,XX,add(5)(q?33)		PB
ANKL2	M	20	Japanese	2004	Alive	yes	yes	yes	yes	no	no	no	positive	PCR	97	13	74	36	83	2	TCRb negative (southern blotting)	46,XY,+8,+X,der(7)t(1;7)(q12;q34)		PB
ANKL3	M	66	Japanese	2012	DoD	yes	yes	yes	no	no	no	no	positive	PCR	72	6,6	10	6	66	NA	TCRb&g both negative (southern blotting)	72~86,XXYY,-Y,-4,-5,add(6)(q21),-7x2,-9,-10,-11,-13,-14,-15,-18,-19,+1~7mar		PB
ANKL4	M	72	Japanese	2011	DoD	yes	yes	yes	yes	no	no	no	positive	PCR	48	5	13	NA	43	NA	NA	47,X,-Y,-8,der(9)t(1;9)(q12;p22),-14,-19,+mar1,+mar2,+mar3,+mar4,+mar5		BM
ANKL5	M	39	Japanese	2014	Alive	yes	yes	yes	yes	no	no	no	positive	PCR	99	6.4	99	94	78	NA	Negative (southern blotting)	normal karyotype		BM
ANKL6	M	18	Japanese	1993	DoD	NA	yes	NA	NA	NA	NA	NA	positive	ISH	pos	neg	neg	neg	pos	NA	NA	NA	9	PB
ANKL7	M	23	Japanese	1992	DoD	NA	yes	NA	NA	NA	NA	NA	positive	ISH	pos	neg	neg	neg	pos	NA	NA	NA	7	PB
ANKL8	F	52	Japanese	1994	DoD	NA	yes	NA	NA	NA	NA	NA	positive	ISH	pos	neg	neg	neg	pos	NA	NA	NA	6	PB
ANKL9	M	55	Japanese	1994	DoD	NA	yes	NA	NA	NA	NA	NA	positive	ISH	pos	neg	neg	neg	pos	NA	NA	NA	6	PB
ANKL10	F	12	Japanese	1995	DoD	NA	yes	NA	NA	NA	NA	NA	positive	ISH	pos	neg	neg	neg	pos	NA	NA	NA	4	PB
ANKL11	M	32	Korean	NA	DoD	yes	NA	NA	NA	NA	NA	NA	positive	ISH	pos	neg	pos	NA	pos	NA	NA	NA		BM
ANKL12	F	81	Taiwanese	2012	DoD	NA	yes	NA	NA	no	no	no	positive	qRT-PCR	pos	neg	neg	pos	pos	pos	polyclonal	80~98,XX,-X×2,+der(2)t(1;2)(q12;q31)×2,+5,-6,+der(6)t(6;?)(q21;?)×4,-7×2,-18,+21×2,+mar×2[cp17]/46,XX[cp3]		PB
ANKL13	F	49	Taiwanese	2015	DoD	yes	yes	yes	yes	no	no	no	positive	qRT-PCR	pos	neg	neg	pos	pos	neg	polyclonal	46,XX,del(13)(q12q14)[cp5]/46,XX,t(9;22)(q34;q11.2)[1]/46,XX[cp14]		BM
ANKL14	F	36	Japanese	2003	DoD	yes	yes	yes	yes	no	no	no	positive	PCR + Southern blotting	99	2	95	80	37	2	TCRb&g both negative (southern blotting)	46,XX,inv(9)(p11q13),add(21)(p11)		PB

F = female
M = male
DoD = dead of disease
NA = not available
BM = bone marrow
PB = peripheral blood
ISH = in situ hybridization
PCR = polymerase chain reaction
qRT-PCR = quantitative real-time polymerase chain reaction
TCR = T cell receptor
*At first visit, tumor cells presented mainly in the spleen. After splenectomy, tumor cell increased in the peripheral blood.
**Immunophenotypes reported as percentages are out of total lymphocyte population, the majority of which represents tumor cells; pos/neg represent tumor cells

Supplementary Table 2. Somatic mutations in protein tyrosine phosphatases in ANKL and NKTCL.

Gene	Amino acid change	Patient	VAF%	SIFT score	SIFT prediction	PolyPhen2 score	PolyPhen2 prediction
PTPRB	Q625K	NKTCL L11	7.1	0.94	Tolerated	0.001	benign
PTPRC	D733del	NKTCL L07	38.3	NA	NA	NA	NA
PTPRF	Y374H	NKTCL L18	17.8	0.14	Tolerated	1	Probably damaging
PTPRM	Q395L	NKTCL L21	8.8	0.22	Tolerated	0.951	Probably damaging
PTPRM	G1017V	NKTCL L15	5.4	0	Damaging	1	Probably damaging
PTPRK	E163K	ANKL4	41.7	0.25	Tolerated	0.999	Probably damaging
PTPRR	V522A	NKTCL L13	30.6	0.04	Damaging	0.917	Probably damaging
PTPRZ1	Y25*	NKTCL L10, L11	6, 7.4	0	Damaging	NA	NA
PTPRZ1	R469H	NKTCL L20	7.2	0.36	Tolerated	0.003	Benign
PTPRO	L959R	ANKL12	22.9	0	Damaging	1	Probably damaging
PTPRT	N421D	NKTCL L12	26.3	0.09	Tolerated	0.995	Probably damaging
PTPN2	R45Q	NKTCL L23	18.75	0.01	Damaging	1	Probably damaging
PTPN3	D226Y	NKTCL L08	36.36	0	Damaging	1	Probably damaging
PTPN4	NA	ANKL12, ANKL13	52.6, 0.1	NA	NA	NA	NA
PTPN13	K2028R	NKTCL L15	4.1	0.54	Tolerated	1	Probably damaging
PTPN13	R147Q	NKTCL L17	1.7	0	Damaging	1	Probably damaging
PTPN21	L472P	ANKL9	43.9	0	Damaging	0.986	Probably damaging
PTPN23	G1061fs	ANKL7	52.6	NA	NA	NA	NA

Supplementary Table 3. Characteristics of NK cell lines used in the study.

Cell line	Diagnosis	Gender	Age at diagnosis	EBV	Reference
IMC-1	ANKL	M	42	negative	Chen I-M, Whalen M, Bankhurst A, Sever CE, Doshi R, Hardekopf D, et al. A new human natural killer leukemia cell line, IMC-1. A complex chromosomal rearrangement defined by spectral karyotyping: functional and cytogenetic characterization. Leukemia Research. 2004 Mar;28(3):275–84.
KHYG-1	ANKL	F	45	negative	Yagita M, Huang CL, Umehara H, Matsuo Y, Tabata R. A novel natural killer cell line (KHYG-1) from a patient with aggressive natural killer cell leukemia carrying a p53 point mutation. Leukemia. 2000.
NK-92	ANKL	M	50	positive	Gong JH, Maki G, Klingemann HG. Characterization of a human cell line (NK-92) with phenotypical and functional characteristics of activated natural killer cells. Leukemia. 1994 Apr;8(4):652–8.
NK-YS	NKTCL	F	19	positive	Tsuchiyama J, Yoshino T, Mori M, Kondoh E, Oka T, Akagi T, et al. Characterization of a novel human natural killer-cell line (NK-YS) established from natural killer cell lymphoma/leukemia associated with Epstein-Barr virus infection. Blood. 1998 Aug 15;92(4):1374–83.
SNK-6	NKTCL	M	62	positive	Nagata H, Konno A, Kimura N, Zhang Y, Kimura M, Demachi A, et al. Characterization of novel natural killer (NK)-cell and gammadelta T-cell lines established from primary lesions of nasal T/NK-cell lymphomas associated with the Epstein-Barr virus. Blood. 2001 Feb 1;97(3):708–13.
NKL	NK-LGL	M	63	negative	Robertson MJ, Cochran KJ, Cameron C, Le JM, Tantravahi R, Ritz J. Characterization of a cell line, NKL, derived from an aggressive human natural killer cell leukemia. Experimental Hematology. 1996 Feb;24(3):406–15.
DERL-7	$\gamma\delta$ cell lymphoma	M	30	negative	Di Noto R, Pane F, Camera A, Luciano L, Barone M, Pardo Lo C, et al. Characterization of two novel cell lines, DERL-2 (CD56+/CD3+/Tcry5+) and DERL-7 (CD56+/CD3-/TCRgammadelta-), derived from a single patient with CD56+ non-Hodgkin's lymphoma. Leukemia. 2001 Oct;15(10):1641–9.
KAI3	CAEBV	M	13	positive	Tsuge I, Morishima T, Morita M, Kimura H, Kuzushima K, Matsuoka H. Characterization of Epstein-Barr virus (EBV)-infected natural killer (NK) cell proliferation in patients with severe mosquito allergy; establishment of an IL-2-dependent NK-like cell line. Clin Exp Immunol. 1999 Mar;115(3):385–92.
YT	ALL with thymoma (cells have NK phenotype)	M	15	positive	Yodoi J, Teshigawara K, Nikaido T, Fukui K, Noma T, Honjo T, et al. TCGF (IL 2)-receptor inducing factor(s). I. Regulation of IL 2 receptor on a natural killer-like cell line (YT cells). The Journal of Immunology. American Association of Immunologists; 1985 Mar 1;134(3):1623–30.

NK-LGL = NK-cell large granular lymphocytic leukemia
CAEBV = chronic active EBV infection
ALL = acute lymphoblastic leukemia
