# Supplementary material for: Aggressive natural killer-cell leukemia mutational landscape and drug profiling highlight JAK-STAT signaling as therapeutic target
Source: Nat Commun. 2018 Apr 19;9:1567. doi: 10.1038/s41467-018-03987-2 (PMC5908809; doi:10.1038/s41467-018-03987-2)
Supplement: Supplementary file 3 — Description of Additional Supplementary Files [file 41467_2018_3987_MOESM3_ESM.pdf]

Supplementary Data

Supplementary Data 1. Summary of software and bioinformatic pipelines used in the study and technical details of sequencing experiments, including assessment of sensitivity and positive predictive value of the tumor-only mutation detection method and sequencing coverage information.

Supplementary Data 2. Somatic mutations identified in 14 ANKL patients by whole-exome sequencing.

Supplementary Data 3. Somatic mutations identified in 25 NKTCL patients by reanalysis of Jiang *et al*. WES data.

Supplementary Data 4. MutSigCV and OncodriveFM analyses of identified mutations in ANKL and NKTCL.

Supplementary Data 5. Expression data, including read counts, $\log_2$ counts per million (CPM), and $\log_2$ reads per kilobase per million (RPKM), of NK cell lines and normal NK cells.

Supplementary Data 6. Mutations in NK cell lines identified by RNA sequencing and targeted DNA sequencing. For cell lines with both data available, presence or absence of mutation in RNA or DNA sequencing data is indicated by 1 or 0, respectively, in the corresponding column.

Supplementary Data 7. Drug sensitivity profiling data, including drug and cell line collections and DSS, IC50, and dose-response curves of NK cell lines and normal NK cells.
